# A Theory of the Pathology of Malarial Hæmaturia

**Published:** 1875-01

**Authors:** Benj. M. Cromwell

**Affiliations:** Albany, Ga.


ATLANTA

MEDICAL AND SURGICAL JOURNAL.

VOL. XII.] JANUARY—1875. [NO. 10.

### Original Communications.

### A THEORY OF THE PATHOLOGY OF MALARIAL HÆMATURIA.

BY BENJ. M. CROMWELL, M.D., ALBANY, GA.

As Dr. Johnson, of Fort Gaines, Ga., in a paper published in the September number of your JOURNAL, has described, in an able and lucid manner, the symptoms, general course and treatment of malarial hæmaturia, I do not deem it necessary to go over the same ground, although dissenting from some of his conclusions, but will confine myself to some reflections on its pathology. I will premise by saying that I advance these views chiefly with the hope that they will be provocative of that discussion which is the touchstone of all theory.

It is evident that, until this and its kindred diseases (the whole malarial group) are made to rest upon a sound pathological basis, their treatment must be empirical. It is only necessary to observe how widely apart are the views of different practitioners, as to the essential remedies to be used in the treatment of this affection, to see the truth of this. While some place their sole reliance upon quinine, others are no loss strenuous in their advocacy of some of the preparations of iron as essential adjuncts, while others base their chief reliance upon it. While some claim opium, blisters, spirits turpentine, etc., as requisite to the successful termination of any case, there are others who contend that their use is at least of doubtful propriety, if not positively injurious.

Now, this confusion could not exist to the extent it does, if there was a well defined idea as to the true pathology of malarial poisoning; and if this paper shall aid in bringing about so desirable a consummation, I shall be well repaid, even should it be at the expense of my own theory.

As illustrating the more aggravated forms of this disease, but chiefly because it was one of only two cases upon which post mortem examinations were held, I will give the history of the case of John Reynolds, alluded to in Dr. Johnson's paper. I did not see the patient during life, but was present, in company with Drs. Hilsman and Miller, at the examination of the viscera, through the courtesy of Dr. Hilsman, the attending physician.

J. R., æt. twenty-four years, resided on the east side of Flint river, in the pine lands, and amongst the ponds. From having repeated attacks of intermittent fever, he became very anæmic—was, in fact, suffering from malarial cachexia. Came to the city on Saturday to attend to some business, and was induced to remain with some friends over night. At eight o'clock A.M. of the next day, was taken with a severe rigor of over an hour's duration, after which he began turning yellow. Was seen by Dr. Hilsman, who administered forty grains of quinine, after which he left, intending to call again at noon. At half-past 12 P.M., had another rigor, in which he expired. *Autopsy*, performed about four hours after death. The skin, fat, and all the tissues were stained a deep yellow; the portal vessels were gorged with fluid blood; the *liver* was enlarged, especially through the antero-posterior diameter of the greater lobe; weighed six pounds and six ounces; was dense and tough in structure, and had the appearance of being fatty. *Spleen* weighed three pounds and one and a half ounces; was of normal consistence and color, and otherwise presented no indications of disease. The *kidneys* were enlarged, weighing five and three-quarters ounces each, but were perfectly healthy in structure, so far as a critical and careful examination without a microscope enabled us to determine; there was no extravasation of blood in or around their substance. The *gall bladder*, when opened, was found to be about half or three-fourths full of bile of the usual consistency, but of a *bright crimson color*, resembling arterial blood as it jets from a freshly cut artery; this color was not affected by its exposure to the atmosphere; when dropped in water, this bile sank to the bottom, developing the characteristic yellow color of bile—and when the water was agitated, it lost entirely its red color, and only the yellow color of bile remained.

I had, in addition to this, notes of another case upon which a post mortem was had, but I had the misfortune to lose or misplace them, and cannot refer to them. I saw the case (J. R. H.) in consultation with Dr. E. L. Connally, now resident in Atlanta, and was present at the autopsy. The chief point of interest that I remember, as occurring during the progress of the case was, that where a blister had been applied, the sacks filled with a bloody serum, resembling in color the urine. The post mortem examination was more negative in this case than in the preceding one, in this, that the liver was found to be not enlarged, or presenting in any way indications of disease. The contents of the gall bladder were, unfortunately, not examined. The spleen and kidneys were enlarged (I do not remember to what extent), but were healthy in structure. There was no extravasation of blood in or around the kidneys.

The failure to find any appreciable change in the liver in this case would seem to indicate that disease in this organ, when found in hæmaturia, is adventitious, and is not a factor entering into its causation.

As regards the scarlet bile found in the gall bladder of Reynolds, I have no explanation to offer. I did hope, at one time, to trace some connection between it and the "bloody urine" of hæmaturia, through a peculiar substance found frequently in the urine of bilious fever patients, which is seen as a pink deposit, clinging to the bottom and sides of vessels that have contained their urine. This substance is purpurine, a carbonized material that appears only in the urine when the kidneys act vicariously for the liver; that organ, as in bilious fever, not performing its functions. Might it not be that the deep red color of hæmaturic urine was due to an excess of purpurine, caused by the total suspension of the function of the liver. I liked this supposition better than the commonly received belief that the color was caused by blood; I could not see how such quantities of blood could escape from the kidneys except by rupture of their capillaries, and consequent disorganization of their structure. For such a supposition the results obtained in the foregoing examinations give no warrant; the structure of the kidneys in both cases was found entire. The only other way in which entire blood corpuscles could be passed through the kidneys was by exuding through the walls of the blood vessels, as in spanæmic diseases, scurvy and purpura. Now, the condition of the serum found in the blistered sacks in the second of the above cases tends strongly to support this view, and I have been told of a case of this disease where blood was said to have oozed from the eyes, nose, mouth and uterus. If this is true, the probability becomes very strong that it is by exudation that the blood is poured out into the bladder.

I am disposed, however, to reject this view of the pathology of hæmaturia, because of the limited number of cases found to sustain it. For even if the above reported case of oozing from the mucous surfaces did occur, the case is exceptional. Out of probably over one hundred and fifty cases of this disease that have occurred in this section of country, I have heard of no other like it. Besides, as I hope will appear as we proceed, I believe a more rational and comprehensive theory may be entertained.

With the view of determining whether or not the color of hæmaturic urine was due to purpurine, Dr. W. W. Bacon, of this city, was kind enough to apply the tests laid down by the authorities in such matters, and obtained negative results; but in testing for blood, he was eminently successful. In addition to this, a sample of hæmaturic urine was forwarded by Dr. Hilsman to Prof. Tyson, of Philadelphia, who stated in reply that the color of the urine was due to broken down blood cells. So the supposition that the urine might owe its color to purpurine was set at rest.

The statement of Prof. Tyson was confirmatory of a view I had previously entertained as to the pathology of this affection, but which the appearance of the bile in the Reynolds case caused me for a time to abandon. It is the view that has prompted this paper—one which I believe contains the germ of the true pathology of all malarial poisoning.

To make it clear as we proceed, let us glance at the *physiological* functions of the blood cells. These little bodies have each, in one sense, an independent existence of its own. They are generated, arrive at maturity, and after fulfilling their destiny, pass away—die. We first meet them, as they emerge from the messenteric glands, as white corpuscles proceeding on their way to the chyle duct, from whence they are thrown into the general circulation, then to perform their allotted task as oxygen and carbonic acid carriers, until their time comes to expire.

We know that the duration of these little corpuscles is limited, because we know that vast quantities of white cells are being elaborated out of the chyle after each meal, and if the process of generation is constantly going on, the process of decay must be as constant.

Now, what becomes of these red corpuscles when they die or are destroyed? Nature must either convey them (or their constituent particles) *out* of the economy, or reconvert them to other uses *in* the economy. For the last supposition, I know of no support. If, then, we concede their elimination, it is of no importance, so far as our theory is concerned, whether the spleen, as Kolliker affirms, is their charnel house, or whether they lose their vitality while running their course in the general circulation; the point that *is* important is, that as soon as they lose their vitality, they become effete matter, and must be thrown out of the economy like all other effete material.

Now, these effete blood corpuscles are composed largely of hæmatin, which is the basis of all coloring matter in the system. This hæmatin is eliminated by the kidneys, the chief great emunctories of the economy.

So far, we have considered the destruction of the red corpuscles as a healthy physiological phenomenon. We see that in health the rate of loss and supply must be in constant proportion. Let us now consider what would be the result if, by the supervention of a disturbing factor, these conditions—this rate—becomes altered.

If red blood is generated faster than it is destroyed, we have plethora—a condition too familiar to be further considered. If, on the other hand, the red corpuscles—these oxygen carriers—while in the performance of their function, come in contact with an agent that is destructive to their vitality (as we suppose malaria to be), we will have presented to us a set of phenomena eminently characteristic of all malarial poisoning.

Let us consider this attentively. The symptom that most strikingly arrests our attention in all cases of paludal poisoning, even the mildest, *is the anæmic condition of the patient.* Robust, red-faced men are as much blanched by one or two paroxysms of an ordinary intermittent as they would be by a serious attack of pneumonia, or other grave disorder not malarial in its character. Todd and Bowman, noticing this fact in their article on the blood (*Pathological Anatomy*), say: "Diminution in the quantity of fibrine, accompanied with a decrease in the red particles, occurs chiefly in fevers arising from the presence of a poison in the system. None show those changes more than those fevers which are caused by the paludal poison—namely, intermittent fever. In rheumatic fever, and in acute general gout, there is a remarkable tendency to the diminution of the coloring matter of the blood, even when the diseases have been treated in the mildest manner. It would seem as if the materies morbi *acted as a blight upon the red corpuscles, and prevented their development* in the normal proportions." [Italics mine.]

I am not certain whether they intend to apply this last observation to the action of poison in paludal fevers, as well as in rheumatism and general gout; but if they do, in ascribing the diminution of the red corpuscles in malarials fevers to an arrest of their formation, instead of to their destruction after they are formed, does not seem to be sustained by clinical observation; for it is well known that often in intermittent fever there is but a slight interruption to the appetite, and none seemingly to the processes of digestion and assimilation. If, then, food is taken and assimilated, blood corpuscles must be formed; but if, after being formed, their integrity is destroyed by being brought in contact with a poison that is being inhaled by the lungs, then may the terms of my proposition be clearly stated as follows:

1. That all malarial diseases, of whatever grade or intensity, are caused by the introduction into the lungs of a specific poison, known as malaria, which, coming in contact with the red corpuscles of the blood, destroys their integrity.

2. That the blood corpuscles so destroyed, being unfit for use in the economy, are eliminated by the kidneys, as effete material.

3. That the severity of malarial paroxysms, is in proportion to the number of blood corpuscles destroyed; and this condition depends upon the intensity of the poison and individual aptitude to its influence.

4. When mild attacks of intermittent fever succeed each other in rapid succession, the destruction of blood disks becomes rapidly greater than their generation, until finally, the vital powers of resistance giving way, or an inordinate dose of the poison being inhaled, a large per centum of the disks are suddenly de-vitalized and malarial hæmaturia is developed by the active exertions of the kidneys to throw off from the system this amount of effete matter.

5. Next to anæmia, the most prominent symptom that shows itself in cases of malarial poisoning is the great prostration of the nervous system. Patients themselves remark this, and say it is so out of proportion to the severity of the attack that they are diposed to attribute it to some other cause.

6. This loss of nerve power is, without doubt, due to malaria-ria, but in a secondary way. I do not believe that it is caused by the poison being brought in direct contact with the nerve centres, but by the diminution in the quantity of blood disks, and the consequent loss of oxygen supplied to the nervous system; for we observe the same phenomena in fasting and other conditions where malaria is not a factor.

7. The greater or less arrest of secretion that we observe in these eases, may be attributed to the loss of that nerve power which stimulates the glandular system to the proper performance of its functions; and it is in some such way as this, I believe, that the formation of the scarlet colored bile in the case of Reynolds is to be accounted for.

I should have stated, when giving an account of the autopsy of Reynolds, that he voided no urine after his seizure at 8 A.M., but when, after death, the bladder was examined, it was found distended with the characteristic "bloody urine."

